# Depressive symptoms and physical activity among community-dwelling perimenopausal women: a prospective longitudinal study

**DOI:** 10.1186/s12888-023-04591-5

**Published:** 2023-02-07

**Authors:** Chuanya Huang, Biru Luo, Jing Wang, Yiling Ao, Weijun Xiong, Shujuan Liao

**Affiliations:** 1 Department of Nursing, West China Second University Hospital, Sichuan University / West China School of Nursing, Sichuan University, #No. 20, Section 3, People’s South Road, Wuhou District, Chengdu City, 610041 Sichuan Province P.R. China; 2 Key Laboratory of Birth Defects and Related Diseases of Women and Children (Sichuan University), Ministry of Education, Chengdu, 610041 China; 3 Ya’an Polytechnic College, Ya’an, 625000 Sichuan China; 4 Sichuan University of Science and Technology, Meishan, 620000 Sichuan China; 5 Chengdu Zhiyong Technology Company Limited, Chengdu, 610041 China

**Keywords:** Women, Perimenopause, Physical activity, Depression, Predictive relationships

## Abstract

**Background:**

Women in perimenopause are vulnerable to depressive symptoms, and physical activity was reported to be a potential protective factor. The trajectories of physical activity and depressive symptoms over time and their longitudinal relationships in Chinese perimenopausal women have not been explored yet, leaving a research gap hindering us from better understanding and managing perimenopause depressive symptoms.

**Methods:**

A multi-center prospective longitudinal study was conducted in four cities in Sichuan Province, China. Depressive symptoms and physical activity in perimenopausal women were collected in March 2019, June 2019, September 2019, and December 2019, respectively. Multivariable linear regression by generalized estimation equation was used to identify the relevant factors associated with depressive symptoms and physical activity. A four-wave autoregressive and cross-lagged panel model was performed to explore their longitudinal relationships.

**Results:**

A total of 1875 women who completed the four-wave data collection were included in the data analysis. Depressive symptoms exacerbated over time and were associated with women’s age, monthly income, marital status, chronic disease, and negative life events. Physical activity decreased over time and was associated with educational background and monthly income. According to the cross-lagged panel model, perimenopausal women with more severe depressive symptoms tended to be less physically active, and similarly, perimenopausal women with less physical activity were more prone to report more severe depressive symptoms.

**Conclusion:**

The cross-lagged panel model disclosed longitudinal bidirectional relationships between depressive symptoms and physical activity in perimenopausal women. Appropriate physical activity should be recommended for perimenopausal women to improve their mental well-being. Tailored physical activity duration and maintenance measures should be proposed based on different sociodemographic statuses.

## Background

Perimenopause is a middle-aged transitional state leading to female reproductive senescence, beginning with variability in the menstrual cycle and continuing to 12 months after amenorrhea [1, 2]. Approximately 88% of women will experience the transition from perimenopause to menopause during their 45–60 years [3]. Women often have physical, psychological, sexual, and behavioral symptoms during perimenopause [4]. Hormonal changes, chronic diseases and some negative life events (e.g., retirement, changing type/conditions at work, and child/family member leaving home) may lead to menopause-related mood disturbances, such as depressive symptoms, even in women without previous history of depressive symptoms [5–7]. Perimenopausal depression and depressive symptoms may also have long-term effects on women, such as sleep disturbance, sexual disturbances, cognitive shifts, and Alzheimer disease [1, 5, 8]. Guidelines for the treatment of perimenopausal depression proposed anti-depressant medication, psychotherapy, and estrogen therapy, which would lead to some severe side effects and economic burdens [5]. Hence, safe and cost-effective interventions to prevent and treat perimenopausal depression are crucial.

Physical activity as a health-related behavior has been widely studied to improve health conditions and quality of life [7]. Several reviews indicated that physical activity has positive effects on preventing depression and relieving depressive symptoms in different age groups [9, 10]. The mechanisms by which physical activity reduces depressive symptoms may include the neurological mechanism (releasing dopamine and improving its effect) and the social mechanism (participation in physical activity improving mastery or sense of coherence) [11, 12]. The relationship between physical activity and depressive symptoms was also reported in previous studies. For instance, a study by Li et al. showed that lower physical activity level was associated with more severe depressive symptoms. Likewise, depressive symptoms could also result in less physical activity among older persons [13]. The physical activity of perimenopausal women may decrease due to menopause-associated hormonal deficiency [14]. A meta-analysis by Faustino R et al. showed that exercise could significantly reduce depressive symptoms in midlife and older women [15]. Additionally, a cross-sectional study by Bondarev et al. showed that a higher level of physical activity was associated with lower mental symptoms in perimenopausal women and suggested that a longitudinal study should be conducted to confirm this finding [16].

Although the necessity of the management of depressive symptoms and physical activity in perimenopausal women was clear, to our best knowledge, no studies have attempted to explore the variation trends of depressive symptoms and physical activity and their interactions among community-dwelling perimenopausal women in China, leaving a research gap hindering health professionals from better understanding and managing perimenopause depressive symptoms. Thus, the objectives of this study were to (1) investigate the longitudinal trajectories of depressive symptoms and physical activity during perimenopause, as well as the relevant factors associated with them among community-dwelling perimenopausal women, and (2) explore the longitudinal bidirectional relationships among depressive symptoms and physical activity.

## Methods

### Study design and settings

A multi-center prospective longitudinal observational study was conducted from March 2019 to December 2019. Participants from four community health centers were selected by convenience sampling. The four community health centers are located in Chengdu, Ya’an, Leshan, and Deyang cities in Sichuan province, China, respectively. Data were collected in the community health centers, the baseline assessment was carried out in March 2019, and follow-up assessments were conducted at a 3-month interval. In this paper, Time 0 (March 2019), Time 1 (June 2019), Time 2 (September 2019), and Time 3 (December 2019) represented the baseline assessment, the first, the second, and the third follow-up, respectively.

Previous studies reported that the mean age of menopause in Chinese women is between 48.94 and 53.6 years [17, 18]. According to the Seventh National Census in Sichuan and *Sichuan Statistics yearbook in 2019* [19], the average monthly income of the Sichuan population was RMB 2100, 13.2% of them had a college education level or above, while 31.3% has a primary education level or below.

### Participants

Women were eligible if they met the inclusion criteria as follows: (1) the Chinese Han nationality, (2) aged 45 ~ 60 years, (3) lived in the participating communities, (4) with variability to the menstrual cycle for at least 12 months, (5) the latest menstruation was within 12 months, (6) with intact uterus, (7) existence of at least one intact ovary, and (9) provided informed consent. The exclusion criteria included: (1) being treated with antipsychotics, and (2) receiving hormone replacement therapy. At each follow-up, we reassessed whether women’s latest menstruation was within 12 months. If it reached 12 months and above, the participant would be excluded from this study. In addition, if women did not complete the four-wave follow-up because of hospitalization for gynecological disorders or receiving therapy for perimenopausal symptoms, they would also be excluded from data analysis.

### Measurement tools

#### Basic information of participants

We used a self-designed form to collect the basic information, including age, educational background (primary and below, junior, senior, and college and above), monthly income (≤1000 Yuan, 1001 to 3000 Yuan, and ≥ 3001 Yuan), marital status (married, divorced, widowed, and single), chronic diseases (yes or no), and negative life events in the past 6 months (yes or no). Chronic diseases were defined as conditions that lasted for at least 1 year and required ongoing medical interventions and/or limited activities of daily living [20]. Life events included the following that had occurred in the past 6 months: death and/or serious illness of a significant other, serious illness of your own, negative events in a relationship, and other experiences of loss.

#### Depressive symptoms

Depressive symptoms were assessed at baseline and at each follow-up. The nine-item Chinese version of the Patient Health Questionnaire (PHQ-9) was used to assess the depressive symptoms of participants. PHQ-9 has been used widely in primary care institutes to screen depressive symptoms and other mental disorders [21–23]. Participants were asked to rate each item on a scale of 0 to 3 based on how much a symptom bothered them in the past 2 weeks (0 = not at all, 1 = several days, 2 = more than half of the days, 3 = almost every day) [23]. The total score of PHQ-9 ranges from 0 to 27, with 0–4 indicates minimal/subclinical depression, 5–9 indicates mild depression, 10–14 indicates moderate depression, 15–19 indicates moderately severe depression, and 20–27 indicates severe depression. Wang et al. evaluated the reliability and validity of the Chinese version of the PHQ-9 in the general population and reported a Cronbach’s alpha coefficient of 0.86 and a test-retest reliability coefficient of 0.86 [24].

#### Physical activity

Physical activity was assessed at baseline and every follow-up. Since no published dedicated tool was found to measure physical activity in perimenopausal women, the Chinese version of the Physical Activity Scale for the Elderly (PASE) was adopted. Being developed by Washburn RA et al. in 1993, PASE is a brief, easy-to-score, reliable, and valid tool for assessing physical activity in community-dwelling adults [25]. It is comprised of 12 self-reported occupational, household, and leisure activity items in the previous 7 days. The total scores ranged from 0 to 793, with higher scores reflecting higher levels of physical activity. PASE is not only applicable to the elderly, it has also been widely used to assess physical activity among adults aged under 60 years [26–28]. Shirley P.C. et al. reported that the Chinese version of PASE demonstrated good test-retest reliability with an intraclass correlation coefficient of 0.81 [29].

### Data collection

Data were collected by registered nurses in the community health centers who were trained by the main researchers of this study. After assessing the eligibility of the participants, the basic information form was completed by data collectors through face-to-face interviews. The questionnaires for depressive symptoms and physical activity were self-reported by participants at baseline and every follow-up. For participants who were difficult in reading and/or writing, data collectors would read the items verbatim and record the answers on their behalf. Ethical approval was obtained from the Institutional Review Board.

### Statistical methods

Only those participants with complete data for four waves were included in the data analysis. IBM SPSS version 25.0 and Mplus 8 were used to perform data analysis. Categorize data was represented with the number (n) and percentage (%). Continuous data were described as mean and standard deviation (SD). Multivariable linear regressions by generalized estimating equations (GEE) were performed to identify factors associated with depressive symptoms and physical activity among perimenopausal women. Pearson correlations were performed to explore the cross-sectional and longitudinal relationships between depressive symptoms and physical activity at baseline and every follow-up. Four-wave autoregressive and cross-lagged panel model in a structural equation modeling framework was conducted to explore the bidirectional relationships between depressive symptoms and physical activity over time after controlling for age, educational background, monthly income, marital status, chronic disease and negative life events of participants. We used the criteria formulated by Hu and Bentler to evaluate the fit of the cross-lagged panel model [30]: Root Mean Square Error of Approximation (RMSEA) below 0.08, Standardized Root Mean Square Residual (SRMR) below 0.08, CFI above 0.95, TLI above 0.9. A two-tailed *p*-value of less than 0.05 was considered statistically significant.

## Results

**Fig. 1:**
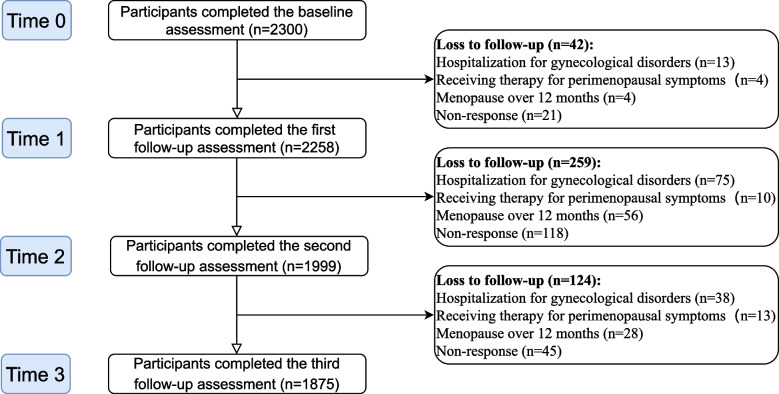
Flowchart of the study participants over the study period

A total of 2300 women were included in this study at Time 0 and 1875 women completed the whole study. Figure 1 showed the details of the drop-out at every follow-up.

### Basic information of participants

Most of the participants aged between 45 and 55 (67.6%), more than half of them had an educational level of less than junior schools (59.3%), less than two-fifths of them were married (39.6%), and the majority of them had a monthly income of more than ¥1001 (77.2%). Almost half of our participants (46.0%) reported chronic diseases, and 38.7% of them reported negative life events. The mean scores of depressive symptoms and physical activity at Time 0 were 2.39 ± 2.64 and 153.12 ± 87.13, respectively. More characteristics of the participants are summarized in Table 1.

**Table 1:** Basic information of participants (*N* = 1875)

Variables	n (%)
Age	
45–50	717 (38.2)
51–55	552 (29.4)
56–60	606 (32.3)
Educational background	
Primary and below	428 (22.8)
Junior	685 (36.5)
Senior	272 (14.5)
College and above	490 (26.1)
Monthly income (¥)	
≤ 1000	429 (22.8)
1001 ~ 3000	995 (53.1)
≥ 3001	451 (24.1)
Marital status	
Married	742 (39.6)
Divorced	262 (14.0)
Widowed	593 (31.6)
Single	278 (14.8)
Chronic diseases	
Yes	863 (46.0)
No	1012 (54.0)
Negative life events	
Yes	725 (38.7)
No	1150 (61.3)
Depressive symptoms, Mean ± SD	2.39 ± 2.64
Physical activity, Mean ± SD	153.12 ± 87.13

### Factors associated with depressive symptoms in perimenopausal women

The GEE model for depressive symptoms showed an increasing trend of depressive symptoms among perimenopausal women over time. Compared with women aged 45–50, those who aged 56–60 had more severe depressive symptoms (B = 0.77, *p* < 0.001). Higher monthly income was associated with lower levels of depressive symptom (B = − 0.86, *p* < 0.001; B = − 0.85, *p* < 0.001). Compared with the married women, those who were widowed and single tended to report higher levels of depressive symptoms (B = 1.11, *p* < 0.001; B = 2.29, p < 0.001). Moreover, chronic disease and negative life events were predictors of more severe depressive symptoms ((B = 1.23, p < 0.001; B = 2.45, p < 0.001) (Table 2).

**Table 2:** Generalized estimation equation for depressive symptoms (*N* = 1875)

Variables	B	SE	*p*-value
Intercept	0.18	0.20	0.363
Time point			
T0	0^a^		
T1	0.46	0.03	< 0.001
T2	0.81	0.05	< 0.001
T3	1.48	0.07	< 0.001
Age			
45 ~ 50	0^a^		
51 ~ 55	0.14	0.12	0.249
56 ~ 60	0.77	0.13	< 0.001
Educational background			
Primary and below	0^a^		
Junior	0.27	0.11	0.068
Senior	1.33	0.22	0.076
College and above	0.29	0.15	0.059
Monthly income			
≤ 1000	0^a^		
1001 ~ 3000	−0.86	0.16	< 0.001
≥ 3001	− 0.85	0.18	< 0.001
Marital status			
Married	0^a^		
Divorced	0.11	0.14	0.467
Widowed	1.11	0.14	< 0.001
Single	2.29	0.23	< 0.001
Chronic disease			
Yes	1.23	0.12	< 0.001
No	0^a^		
Negative life events			
Yes	2.45	0.13	< 0.001
No	0^a^		

Note: 0^a^: reference

### Factors associated with physical activity in perimenopausal women

According to our GEE model for physical activity, the physical activity of perimenopausal women lessened over time. Compared with the women with primary and below educational backgrounds, the women with junior educational backgrounds showed lower physical activity (B = − 13.03, *p* = 0.013). Women with a monthly income of over ¥3001 were associated with a higher level of physical activity compared to women with a monthly income below ¥1000 (B = 14.479, *p* = 0.039) (Table 3).

**Table 3:** Generalized estimation equation for physical activity (*N* = 1875)

Variables	B	SE	*p*-value
Intercept	15.56	3.70	0.078
Time point			
T0	0^a^		
T1	−3.75	0.50	< 0.001
T2	− 6.65	0.67	< 0.001
T3	−7.60	0.74	< 0.001
Age			
45 ~ 50	0^a^		
51 ~ 55	4.13	4.76	0.249
56 ~ 60	−5.39	4.68	0.385
Educational background			
Primary and below	0^a^		
Junior	−13.03	5.25	0.013
Senior	−12.49	6.82	0.067
College and above	0.743	6.07	0.903
Monthly income			
≤ 1000	0^a^		
1001 ~ 3000	4.36	4.93	0.377
≥ 3001	14.479	7.01	0.039
Marital status			
Married	0^a^		
Divorced	15.85	6.67	0.068
Widowed	9.20	6.10	0.130
Single	−0.90	7.18	0.900
Chronic disease			
Yes	−6.56	4.68	0.161
No	0^a^		
Negative life events			
Yes	−5.98	4.93	0.225
No	0^a^		

Note: 0^a^: reference

### The relationships between depressive symptoms and physical activity

**Fig. 2:**
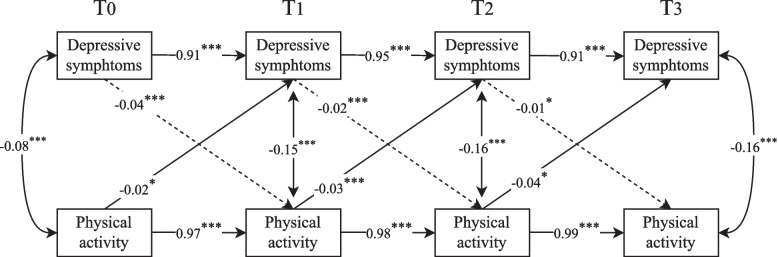
The cross-lagged panel model between depressive symptoms and physical activity. The single arrow represents the regression path and the double arrow represents the correlation. The path coefficient reflects the standardization of the model estimation beta value. Age, educational background, monthly income, marital status, chronic disease, and negative life events were entered in this model as covariates, but they are not described for clarity. (***p < 0.001; **p < 0.01; *p < 0.05; T: Time)

The results of Pearson correlations showed that the depressive symptoms in perimenopausal women at baseline and every follow-up were negatively associated with physical activity accordingly (Table 4). Hence, a cross-lagged panel model was merit to proceed. The goodness of the model fit of is acceptable, with CFI = 0.988, TLI = 0.973, SRMR = 0.012, and RMSEA = 0.021. The cross-lagged panel model showed that a significant cross-lag effect links more severe depressive symptoms with lower levels of physical activity, and vice versa. Therefore, a reciprocal relationship exists between depressive symptoms and physical activity from baseline assessment to our last follow-up in perimenopausal women (Fig. 2).

**Table 4:** Descriptive statistics and bivariate correlations of study variables (*N* = 1875)

Variables	Mean ± SD	1	2	3	4	5	6	7	8
1. Depressive symptoms at T0	2.39 ± 2.64	1.00							
2. Depressive symptoms at T1	2.86 ± 3.32	0.91^***^	1.00						
3. Depressive symptoms at T2	3.20 ± 3.69	0.85^***^	0.96^***^	1.00					
4. Depressive symptoms at T3	3.87 ± 4.49	0.74^***^	0.84^***^	0.92^***^	1.00				
5. Physical activity at T0	153.12 ± 87.13	− 0.10^***^	− 0.09^***^	− 0.11^***^	− 0.11^***^	1.00			
6. Physical activity at T1	149.36 ± 85.65	− 0.11^***^	− 0.14^***^	− 0.16^***^	− 0.17^***^	0.97^***^	1.00		
7. Physical activity at T2	146.50 ± 84.56	− 0.12^***^	− 0.16^***^	− 0.19^***^	−0.20^***^	0.94^***^	0.98^***^	1.00	
8. Physical activity at T3	145.52 ± 85.01	−0.12^***^	−0.15^***^	− 0.18^***^	−0.21^***^	0.93^***^	0.97^***^	0.99^***^	1.00

*T* Time

^***^P < 0.001

^**^p < 0.01

^*^p < 0.05

## Discussion

In this study, we collected the four-wave depressive symptoms and physical activity among community-dwelling perimenopausal women in four cities in Sichuan Province, China. Although the depressive symptoms in perimenopause have been repeatedly observed in different study samples, this is the first multi-centered longitudinal study conducted among the community-dwelling perimenopausal women in southwest China, collecting both depressive symptoms and the physical activity of perimenopausal women as well as making effort to explore their longitudinal relationships. Our results showed that depressive symptoms in perimenopausal women exacerbated over time, while the level of physical activity decreased over time. The longitudinal bidirectional relationships between depressive symptoms and physical activity have been disclosed by using a cross-lagged panel model. In addition, we found that age, monthly income, marital status, chronic disease, and negative life events were associated with depressive symptoms, while educational background and monthly income were associated with physical activity in perimenopausal women. The findings of this study would add data to the literature on perimenopausal depressive symptoms and physical activity, and can help health professionals better manage depressive symptoms in perimenopause women and relieve long-term burdens.

The exacerbating trend of depressive symptoms among perimenopause women in our study is in line with previous findings, which stress that perimenopause is a vulnerable phase to suffer depressive symptoms [31, 32], appealing to special attention to this fragile but inevitable stage in women’s lives. Through the generalized estimation equation, we found that some sociodemographic variables were associated with depressive symptoms. The older the age, the more severe the depressive symptoms in perimenopausal women, which is consistent with the study findings of H-L Lin et al. [33] and Anouk E. de Wit et al. [34]. Compared with the women whose marital status was married, those with single and widowed marital status reported more severe depressive symptoms, which may be related to loneliness in single and widowed status [35]. Kendler et al. [36, 37] also revealed that negative life events and chronic diseases were associated with perimenopausal depressive symptoms, which supported our findings. Additionally, lower monthly income was found associated with more severe depressive symptoms in this study and can be explained by the interrelation between economic and health status. These findings remind community health professionals that more attention should be paid to the single and widowed perimenopausal women aged 56 ~ 60, with lower monthly incomes, and having negative life events and chronic diseases.

Physical activity in perimenopausal women was found to decline gradually during the three follow-ups in the present study. Previous studies by Dmitriy Bondarev et al. [38] and Kurina, L. M. et al. [39] also reported a downward trend in physical activity in perimenopausal women. From the multivariable regression model for physical activity, women with junior or senior educational backgrounds were prone to have lower levels of physical activity, while higher monthly income was associated with higher levels of physical activity. Hence, the benefits of physical exercise and a healthy and active lifestyle should be stressed by health professionals, especially for menopausal women with lower monthly income and lower educational backgrounds.

From the cross-lagged panel model, we found that higher levels of depressive symptoms in perimenopausal women could predict the subsequent lower levels of physical activity, and vice versa. Several studies found that physically active perimenopausal women were more likely to report higher life satisfaction and better mental welling-being [40, 41]. Grindler NM et al. also pointed out that long-term physical exercise was associated with reduced adverse mood [42]. Our findings added information to the published literature by unveiling the cross-lag effect between depressive symptoms and physical activity in perimenopausal women. From the findings of our study, we recommend that perimenopausal women with depressive symptoms should strengthen exercise to alleviate the negative symptoms and those without depressive symptoms should also keep appropriate physical activity to prevent themselves from depressive symptoms.

Our study also has several limitations. First, the follow-up in this study only lasted for 9 months because of the restricted budget and resources. Future studies with longer follow-ups should be considered to further understand how depressive symptoms and physical activity alter the entire perimenopause. Second, the four-time points in which data in this study were collected represent the four seasons of the same year, and thus may have potential seasonal effects on both physical activity and depressive symptoms. Third, as mentioned in the methods section, there is no targeted assessment instrument for perimenopausal physical activity, we used the PASE as a replacement. In addition, this study only included sociodemographic information as influential factors of depressive symptoms and physical activity, and more factors, such as lifestyle factors, could be considered in the future. Last, there was an unavoidable sampling error as this study was a convenience sample in four communities. Though the existence of previous examples and the validation of PASE in the general population, a specific measurement tool for perimenopausal women’s physical activity deserves more effort.

## Conclusions

The cross-lagged panel model disclosed longitudinal bidirectional relationships between depressive symptoms and physical activity in perimenopausal women. Appropriate physical activity and an active lifestyle should be recommended for perimenopausal women to improve their mental well-being. Tailored physical activity duration and maintenance measures should be proposed based on different sociodemographic statuses.

## Data Availability

The datasets used and/or analysed during the current study are available from the corresponding author on reasonable request.

## Abbreviations

PHQ-9: The 9-item of Patient Health Questionnaire
PASE: Physical Activity Scale for the Elderly
SD: Standard deviation
GEE: Generalized estimating equations
RMSEA: Root Mean Square Error of Approximation
SRMR: Standardized Root Mean Square Residual
